# Changes in the West African forest-savanna mosaic, insights from central Togo

**DOI:** 10.1371/journal.pone.0203999

**Published:** 2018-10-05

**Authors:** Honam Komina Atsri, Yawo Konko, Aida Cuni-Sanchez, Komla Elikplim Abotsi, Kouami Kokou

**Affiliations:** 1 Laboratoire de Recherche Forestière, Faculté des Sciences, Université de Lomé-Togo, Lomé, Togo; 2 Agence Nationale de Gestion de l’Environnement, Ministère de l’environnement et des ressources forestières du Togo, Lomé, Togo; 3 Environment Department, University of York, Heslington, York, United Kingdom; West African Science Service Centre on Climate Change and Adapted Land Use/Research Department, BURKINA FASO

## Abstract

The West African forest-savanna mosaic, an important habitat for biodiversity and humans, is severely degraded, fragmented and modified by human activities. However, few studies have quantified the land cover changes observed over time and/or analysed the drivers of change. This study focused on Fazao-Malfakassa National Park, the largest in Togo, uses a combination of remote sensing, ground surveys and questionnaires to: (i) quantify vegetation changes, (ii) determine the drivers of change, (iii) compare results with findings elsewhere in the region and (iv) suggest management interventions. The images used were Landsat 5 TM, Landsat 7 ETM and Sentinel-2. Different vegetation indices were computed including: number of fragments, index of dominance, mean area of a vegetation type and mean annual expansion rate. In total, 300 people (including park staff and local populations) were interviewed using a semi-structured questionnaire. Results indicate that between 1987 and 2015 closed-canopy forest and tree-savanna became severely degraded and fragmented, following trends in other parts of the West African forest-savanna mosaic. The main drivers of change were agricultural expansion, bush fires and timber extraction. Observed changes and drivers altered with time: e.g. agricultural expansion was greatest during 1987–2001 (linked with political instability) while illegal timber extraction augmented during 2001–2015 (following increased timber value). Park staff and local populations’ perceptions on drivers of change did not differ. Our study highlights that action is urgently needed if we are to preserve this important habitat, the biodiversity it hosts and the services it provides to humans. We suggest several management interventions, learning from successful interventions elsewhere in the region.

## Introduction

The West African region, about 6 million km^2^, is characterised by a wide range of ecosystems, related to differences in climate and topography. They include rainforests (1500–3000 mm annual rainfall), forest-savanna mosaic (1200–1500 mm), savannas (800–1200 mm), the Sahel (200–500 mm) and desert (<200 mm) [1–3].

The forest-savanna mosaic, located between 2 and 10 degrees north latitude [4], has an area of 673,000 km^2^ [5]. This includes different types of savannas, open and closed-canopy forests. The forest-savanna mosaic is the preferred habitat for several large mammals (elephants, buffalos, hartebeest), primates and birds [6] and it provides local communities with arable land, wild fruits and honey, bush meat, firewood, building materials, water and grazing areas, among other regulatory and cultural ecosystem services [7].

Unfortunately, these forest-savanna mosaics have been severely degraded, fragmented and modified by human activities, such as slash-and-burn agriculture, mining, unsustainable harvesting of wild resources and urbanisation [8]. Climate change poses another threat to these ecosystems [9]. Regional climate models predict increased temperatures and reduced rainfall for the forest-savanna mosaic [10]. Recent findings from the PARCC project (Protected Areas Resilient to Climate Change) highlighted that 584 bird species of the 1172 studied are sensitive to predicted changes in climate [11].

Protected areas are the best tool we have to conserve biodiversity and ecosystems [8]. Unfortunately, in West Africa, protected areas are under increasing human pressure. The situation is particularly alarming in Togo, because of increasing population (2.7% [12]), poverty (Togo is classed 162 over 167 countries [13]), development needs and past political history.

Togo is one of the smallest countries in Africa (56,600 km^2^) but it has over 83 protected areas which cover 14% of the territory [14]. In the past, protected areas were extended in an authoritarian manner with no compensation paid to local residents, leading-in most cases- to extensive displacement of communities. During the socio-political problems of 1990–1993, some communities settled within certain protected areas (sometimes constituting entire villages), while looking for land for agriculture and grazing. The resulting destruction of natural habitats is the main cause of the degradation observed in most protected areas in Togo [15]. For most of the protected areas management needs improving [16]. Agricultural encroachment and illegal exploitation of resources can severely affect ecosystem functioning. Yet, their negative effects on the ecosystem can be reduced if trends and drivers of change are understood [17].

The Fazao-Malfakassa National Park, located within the forest-savanna mosaic, is the largest national park in Togo, and one of the most important protected areas in this country [14]. In 2012, it was added to the UNESCO World Heritage Site Tentative List, in the mixed (cultural + natural) category. It has an outstanding biodiversity, which comprises 19 large mammals, six primates and 201 species of birds, including *Fraseria cinerascens*, a new record for Togo [18–20]. This national park is threatened by numerous illegal activities including: hunting, cattle grazing, timber exploitation, bush fires and agricultural encroachment [18].

The impacts of these illegal activities on the different habitats have not been evaluated. Yet, this information would help assess vegetation changes over time [21–23] and its impacts on the distribution of wildlife [24]. Although a number of studies have been carried out [25–27], they do not provide enough information to specifically make suggestions for improved management strategies. In order to fill in this gap, it is important to have accurate data on the types of habitats present in the park and their areas, as well as on changes on their fragmentation and degradation over time [28,29]; information which could be used to help preserve the complexity, integrity and representativeness of each habitat type. This type of studies can also help identify the causes of the observed changes so that adaptive management interventions can be proposed [30]. Although a study on land use change on the Mono River Basin of central Togo (including the northern part of Fazao-Malfakassa National Park) has recently been published [31], the study analysed few land use classes (and used few geo-referenced locations to determine the land use classes and changes, which questions its accuracy) and it did not assess drivers of change.

The aim of this study, focused on Fazao-Malfakassa National Park in Togo, was to (i) quantify vegetation changes observed between 1987 and 2015, (ii) determine the drivers of change, (iii) compare results with findings elsewhere in the region, and (iv) suggest management interventions. Through this case study in Togo, we aim at highlighting trends in the West African forest-savanna mosaic. The approach presented here to assess vegetation changes and the drivers of change is easily replicable and could be used to help inform management decisions elsewhere in the region.

## Study area

**Fig 1:**
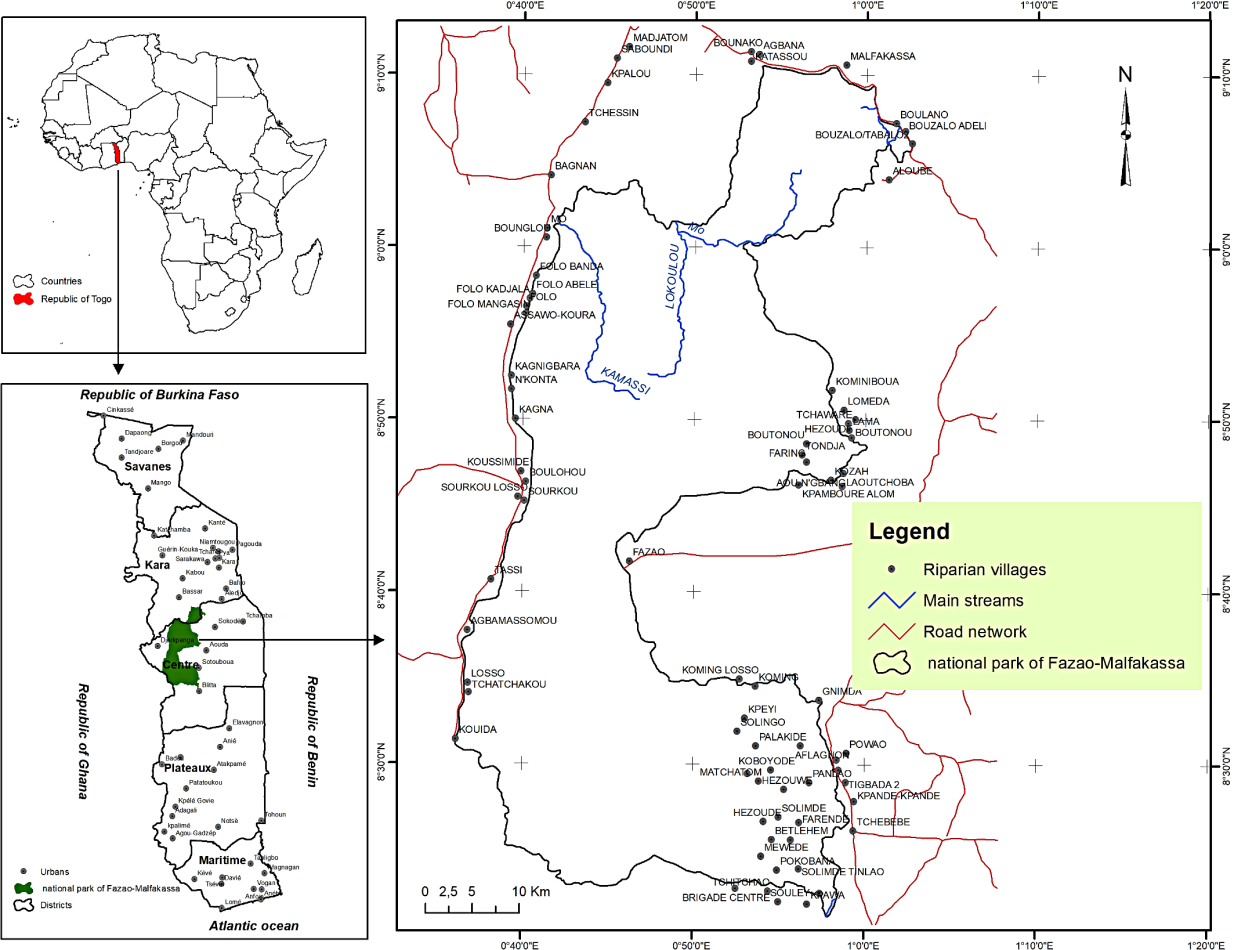
Location of Fazao-Malfakassa national park in central Togo, West Africa.

This study focused on Fazao-Malfakassa National Park (thereafter called FMNP), which is situated in central Togo, at the limit between the Sudanian and the Guinean ecological zones (Fig 1). The FMNP comprises Mt Fazao (861 m), Mt Malfakassa (713 m) and plains where Mo River flows. These mountains are part of the Atacora chain, which was formed during the pan African orogenesis [32]. The region has a unimodal rainfall regime, with most rains falling in April-October. Annual rainfall ranges between 1200 and 1500 mm. Mean temperature during the rainy season is 25°C, while temperature during the dry season ranges between 15 and 40°C [33]. Mountains and hills have lithosols while the plains have sandy and sandy-clay soils [34].

FMNP was created in 1975, when Fazao Forest Reserve (162,000 ha) and Malfakassa Forest Reserve (30,000 ha) were combined. FMNP was manged by the Ministry for the Environment and Forestry Resources (MERF in French) up to 1990, by Franz Weber Foundation between 1990 and 2015, and by MERF afterwards. In 2012, FMNP was added to the UNESCO World Heritage Site Tentative List, but this addition has not been formalised yet. In 2014, with the aim of reducing agricultural encroachment, a number of poles were set up on the south-eastern part of the park. These poles were located within the park, at the edge of the agroforestry habitat, and are not representative of the official park limits.

The park is surrounded by several villages, which are from Tem, Agnanga, Adélé, Bassar and Kabyè ethnic groups [35]. In 2010 a census estimated that the surrounding population of the park was about 120,000 people [36]. In this region, local communities practice small-scale subsistence slash-and-burn agriculture: a piece of land is cleared (first trees are cut and then remaining vegetation burned), it is cultivated for a number of years, and when soil fertility decreases, it is abandoned (and a new piece of land is cleared). Local communities usually prefer to clear tree savanna as it is believed that its soil is more fertile. The crops cultivated are yam, maize, groundnuts, cowpeas and cotton. Local communities do not cut important fruit trees such as *Parkia biglobosa* and *Vitellaria paradoxa* when land clearing, as reported from other areas in West Africa [37].

In this park, hunting, timber and firewood collection, charcoal production, grazing of livestock and harvesting non-timber forest products are illegal activities. There are no major cities or tarmacked roads around the park, except for the north boundary (Sokodé-Bassar road, see Fig 1). Few untarmacked motorable roads cross the park, mainly in the south-eastern part. The FMNP has a fire management plan. In order to promote fresh pasture for wildlife, park rangers burn the savannas in the park at the onset of the dry season (late November). As this is not carried out in a very controlled manner, fire might reach open forest, but fires are not aimed for this vegetation type.

Several large mammals are found in FMNP, including savanna and forest elephant (*Loxodonta africana* and *L*. *cyclotis*, VU and EN respectively), hartebeest (*Alcelaphus buselaphus*, LC), African buffalo (*Syncerus caffer*, LC) and lion (*Panthera leo*, VU) [18] (unpublished data for lions). Endangered statuses follow the IUCN Red List: EN: endangered, VU: vulnerable, LC: least concern (see http://www.iucnredlist.org/). Important primates include: *Colobus vellerosus* (VU), *Perodicticus potto* (LC), *Galago senegalensis* (LC) and *Cercopithecus mona* (LC). Large forest birds include *Accipiter erythropus* (LC) and *Tauraco persa* (LC) [19]. The FMNP also has several threatened tree species including: *Afzelia africana* (VU), *Cordia platythyrsa* (VU), *Khaya senegalensis* (VU), *K*. *grandifoliola* (VU), *Pararistolochia goldieana* (VU), *Pouteria alnifolia* (VU) and *Vitellaria paradoxa* (VU) [18] (S5 Table in supporting information).

A research permit was obtained from the Ministry for the Environment and Forestry Resources (MERF) and by the director of the national park. We confirm that the field studies did not involve endangered or protected species.

## Data and methods of data analysis

### Satellite image analysis

Landsat 5 TM (23/01/1987, 7 bands, 30 m resolution), Landsat 7 ETM (7/12/2001, 9 bands, 30 m resolution) and Sentinel-2 (21/12/2015, 13 bands, 10 m resolution) were used in this study. They were obtained from https://landsat.usgs.gov/ and https://sentinel.esa.int/ respectively. The path/row of the Landsat images is 193/054. While a single Landsat image covered the whole of our study area, two Sentinel-2 tiles had to be mosaic. All images are considered of high resolution and their comparison is widely accepted [38]. We choose Sentinel-2 images instead of Landsat 8 because the first had fewer clouds than the latter for our study region.

Images were corrected geometrically (DN to surface reflectance) and were enhanced by levelling histograms of the different channels (to reduce contrasts) using the Gaussian filter. TM and ETM images were reclassified into 10 m resolution using the algorithm PC Spectral Sharpening tool of ENVI 4.3. For each image (Landsat TM, ETM and Sentinel-2), we combined three bands to generate composites of natural colours. For TM and ETM, band 5 (short-wave infrared; 1.55–1.75 μm, associated with the red channel), band 4 (near infrared; 0.76–0.9 μm, associated with the green channel) and band 3 (red; 0.63–0.69 μm, associated with the blue channel) were selected for visual interpretation and creation of ROI (land use classes) while for Sentinel-2, band 4 (red; 0.665 μm), band 2 (blue; 0.49 μm) and band 3 (green; 0.56 μm) were selected.

We used 112 geo-referenced records, collected in December 2016, as ROI to train a supervised classification. These included: 15 in shrub savanna, 18 in tree savanna, 20 in savanna-woodland, 20 in open forest, 21 in closed-canopy forest and 18 in agroforestry. Google Earth and an old survey by FFW [18]) were also used to assess the classification. The supervised classification we performed with the ROIs used a maximum likelihood algorithm. This approach is based upon the Bayes theorem which allows for the description of classes within an image depending upon probability density [39]. It is a method widely used for this purpose [40,41].

The vegetation categories for the ROIs were determined using the dominant tree species in each vegetation type [18]. The classes followed Yangambi vegetation nomenclature [42] which includes: shrub savanna, tree savanna, savanna-woodland, open forest, closed-canopy forest and agroforestry (see S1 Table in supporting information). Closed-canopy forest refers to semi-deciduous forest, dry deciduous forest and riparian forest. Open forest might also be defined as woodlands but we prefer to use the term open forest to avoid confusion with savanna-woodland. Agroforestry comprises agroforestry, crop fields and abandoned fields (fallows).

After image classification of the Sentinel-2 images of 2015, a field survey (January 2017) was used to verify the vegetation types on the ground. For this purpose, 240 geo-referenced locations, randomly chosen using ArcGIS, were selected. These included: 32 in shrub savanna, 37 in tree savanna, 52 in savanna-woodland, 45 in open forest, 40 in closed-canopy forest and 34 in agroforestry. A confusion matrix was used to assess the percentage of correctly classified points and Kappa coefficient was used to determine classification accuracy of the Sentinel-2 images of 2015 (see S2, S3 and S4 Tables in supporting information for further details).

Landis and Koch [43] scale was also used to determine if our images were accurately classified (classes are: very good, good, moderate and bad, when Kappa coefficient is >80%, 60–79%, 40–59% and <40% respectively). All images were transformed from raster to vector to create polygons, so that number of polygons and the area of each polygon could be estimated. All analyses were performed in ENVI 4.3 (image analysis) and ArcGIS 9.3 (vectorisation and estimation of polygons’ area).

### Assessment of landscape change over time

Four indices were computed to determine vegetation changes over time: number of fragments, index of dominance, mean area of a vegetation type and mean annual expansion rate [44,45]. The number of fragments of a vegetation type (*N*_*i*_) refers to the number of polygons of this vegetation type in a given date. The index of dominance Dj *(a)* (expressed in %) refers to the proportion of area occupied by the largest fragment with regard to the total area occupied by a given vegetation type *j* [46].

$$D_{j}(a)=\frac{SPmaxj}{atj}\times 100$$

where Sp_maxj_ refers to the area of the largest fragment of given vegetation type and at_j_ to the sum of all fragments’ areas of the same vegetation type. The index Dj (a) ranges between 0 and 100. The lower the value, the more fragmented is a given vegetation type [46]. The mean area of a vegetation type $\bar{\alpha }$_j_ (expressed in %) is calculated as follows:

$${\bar{\alpha }}_{j}=\frac{\mathrm{atj}}{\mathrm{Nj}}$$

where *at*_*j*_ refers to the sum of all fragments’ areas of the same vegetation type and Nj to the number of fragments of a given vegetation type j.

The mean annual expansion rate (*T*) (expressed in %) [47–49] was used to assess changes in areas between years (1987, 2001 and 2015). This index was calculated as:

$$T=\frac{\ln\left(S2\right)-\ln\left(S1\right)}{t*ln(e)}\times 100$$

where S1 and S2 refer to the area of a given vegetation type in the first and second date considered (respectively), and *t* refers to number of years between the first and second date considered (note that ln = natural logarithm and e is a constant = 2.71828).

### Drivers of change and literature review

In order to determine the factors which caused the observed changes in the landscape, 300 people were interviewed using a semi-structured questionnaire. These included eight park managers (former or current) (35–55 years old), 38 park rangers (20–45 years old), 88 male farmers (20–60 years old), 52 female farmers (20–60 years old), 72 male hunters (20–60 years old), and 42 young people (16–19 years old), which lived in 22 villages around the park. They were selected on a voluntary basis, they were not paid for participating in the study and they were first informed of the aim of the study. In the villages, we first explained the aim of the study to the village chief, and the number and type of participants we needed. He/she then asked some residents to participate. The interviews were facilitated and translated by a person of the same ethnicity of the village we were working on.

Participants were asked to identify major and minor drivers of land cover change from a list provided by the interviewer. The drivers considered included: clearing land for agriculture, charcoal production, bush fires, timber extraction, livestock grazing, firewood collection, honey collection, wild fruits’ collection and hunting. Bush fires refers to fires lighted by hunters at the end of the dry season (from January onwards), which tend to be more destructive than the fires used by park rangers in the annual fire management plan (which take place in late November). These bush fires set up by hunters might even reach closed-canopy forest (pers. Obs.). We also asked participants to determine in which habitat type these activities were carried out and which tree species were targeted for the different activities (timber, firewood, charcoal and wild fruits).

Drivers’ relative importance (N_i_) was calculated [50] as follows:

$$N_{i}=\frac{Nf}{Nt}x\;100$$

where N_f_ refers to the number of respondents mentioning this driver as major driver and N_t_ refers to the total number of respondents. Results on drivers of change are reported (a) combining all respondents’ answers, and (b) grouping respondents’ answers into two groups: park staff (managers and rangers) and local populations. In this latter case, a t-test was used to determine significant differences between groups.

In order to determine if trends in land use change and drivers of change in our study area followed those observed elsewhere in the West African forest-savanna mosaic, we conducted a literature review. We used Google Scholar and the following key words (in English and French): forest-savanna mosaic, forest, savanna, West Africa, drivers of change, Benin, Togo, Ghana, Mali, Niger, Burkina Faso, Ivory Coast, Senegal. Studies which only focused on closed-canopy rainforest were excluded from the review. We did not include any date range limit when searching for publications; and we included studies in both protected and non-protected areas. The search was conducted in August 2017. For each study reviewed, we recorded: the methodology used, the changes reported in each land use category (increase, decrease and no change) and the drivers of change. The location of all the studies reviewed can be found in S1 Fig in Supplementary information.

## Results

### Observed changes in the landscape

Results indicate that the six land cover types studied were accurately classified (rated very good in the Landis and Koch scale). Kappa coefficients were >80% for the three images we analysed (see S2, S3 and S4 Tables in supporting information). However, it should be mentioned that accuracy decreased for the 2015 image. This is likely to be related to increased habitat fragmentation: e.g. small fragments of tree savanna embedded within agroforestry, which were miss-classified as agroforestry).

**Fig 2:**
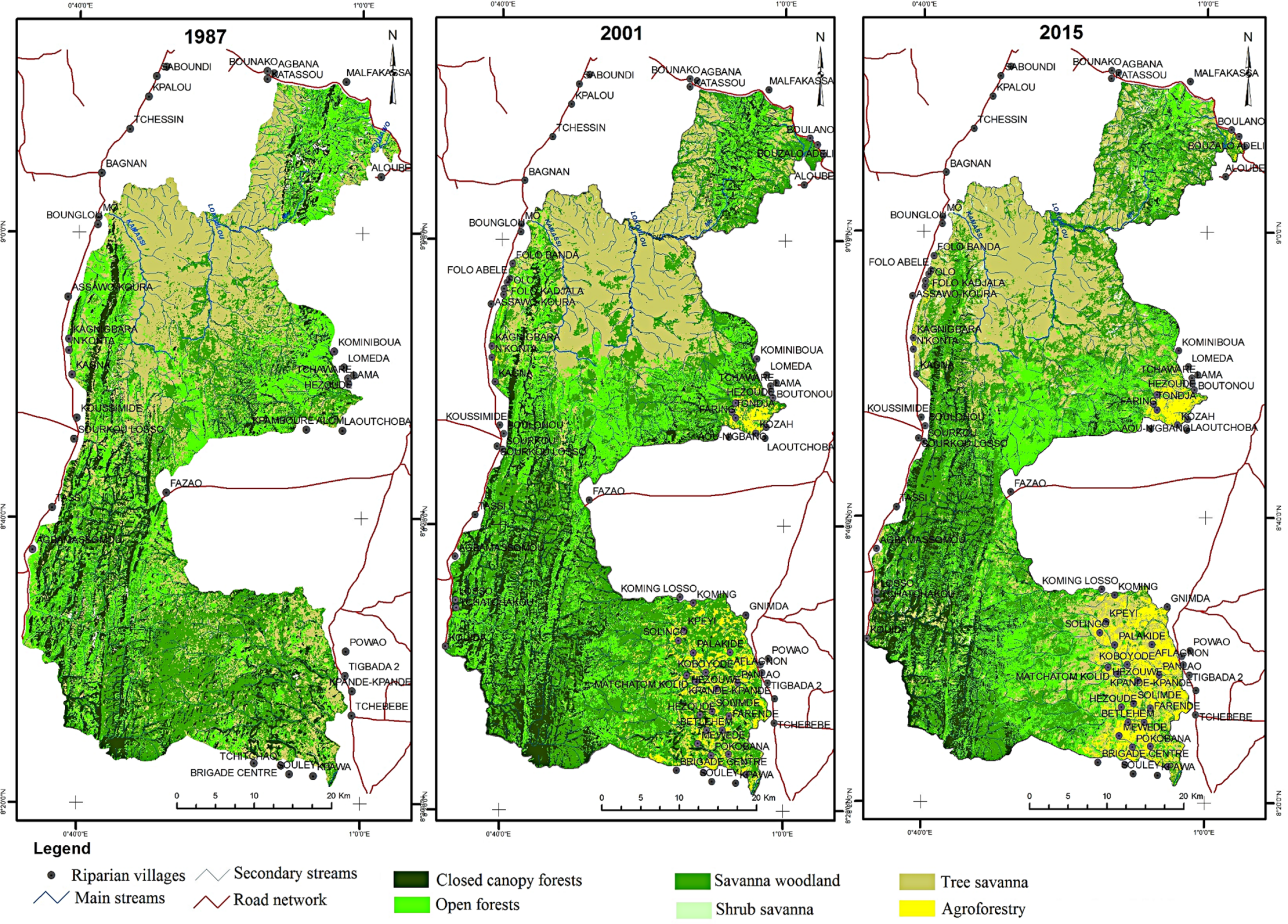
Trends in land cover and land use between 1987 and 2015. AG: Agroforestry; SS: Shrub Savanna; SW: Savanna Woodland; TS: Tree Savanna; OF: Open Forest; CCF: Closed-Canopy Forest.

In 1987 the dominant vegetation types were found to be (in decreasing order of area): tree-savanna, open forest, savanna-woodland, closed-canopy forest, shrub savanna and agroforestry (Fig 2). Between 1987 and 2015 the area of closed-canopy forest and tree-savanna decreased (40% and 20% decrease respectively). The decrease in closed-canopy forest was greater in 2001–2015 than inn 1987–2001, during which they slightly increased in area (3.48%, see Table 1). This is likely to be explained by the fact that before 2001, park management was highly repressive, in a military manner, which limited illegal activities. However, after 2001, park management became less repressive and illegal activities increased.

**Table 1:** Percent of change and annual expansion rate between 1987 and 2015 for the different vegetation types studied.

Land cover	Percent of change			Mean annual expansion rate (%)	
	1987–2001	2001–2015	1987–2015	1987–2001	2001–2015
**CCF**	3.48	-43.08	-41.1	0.05	-0.76
**OF**	1.18	0.30	1.48	0.01	0.00
**SW**	3.41	17.82	21.84	0.04	0.22
**TS**	-15.44	-5.39	-20.00	-0.21	-0.07
**SS**	45.45	82.47	165.4	0.47	0.81
**AG**	37025.74	118.83	8 1141.26	7.44	1.06

AG: Agroforestry; SS: Shrub Savanna; SW: Savanna Woodland; TS: Tree Savanna; OF: Open Forest; CCF: Closed-Canopy Forest.

**Fig 3:**
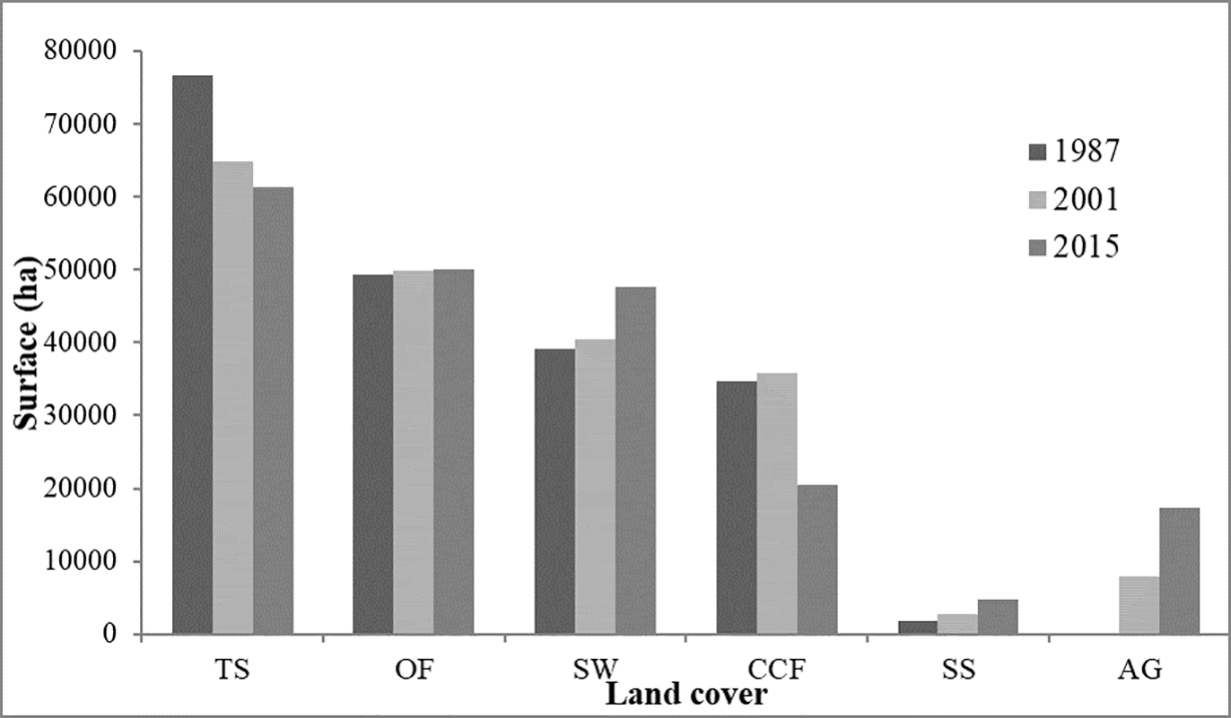
Vegetation types in 1987 (left) 2001 (center) and 2015 (right) with regard to the different years studied.

Between 1987 and 2015, the extent of agroforestry, shrub savanna and savanna-woodland increased; and that of open forest barely changed (Fig 2, Table 1). The rates of change were similar between both time periods for most vegetation types except agroforestry, for which it was greater for the period 1987–2001. While in 1987 there was more area of closed-canopy forest than of agroforestry, in 2015 their areas were similar, about 20,000 ha (Fig 2). The expansion of agroforestry was mainly located at the south-eastern and central-eastern part of the park, where there are more villages (Fig 3). The western part of the park, which is more mountainous, experienced fewer changes.

Over time, habitat fragmentation increased in all vegetation types. The increase was particularly important for closed-canopy forest (from 7,000 to 32,000 fragments). The mean area of all vegetation types (except agroforestry) decreased over time. The dominance index decreased for closed-canopy forest, tree-savanna and shrub savanna, while it increased for the other vegetation types (Table 2).

**Table 2:** Trends in spatial structure indices for the different vegetation types and years.

Indices	CCF	OP	SW	TS	SS	AG
**n1987**	7 041	8 668	14 481	14 432	908	9
**Dj(a)1987**	3.85	4.64	3.23	32.61	0.20	17.30
$\bar{\alpha }j1987$	4.72	5.69	2.71	5.32	2.00	2.37
**n2001**	6 981	13 529	19 049	26 725	1 353	1 597
**Dj(a)2001**	4.12	6.11	4.12	31.15	0.20	1.20
$\bar{\alpha }j2001$	5.14	3.69	2.13	3.73	1.95	4.96
**n2015**	32 032	16 071	24 686	33 920	6 352	3 311
**Dj(a)2015**	2.63	7.68	4.79	28.13	0.15	1.37
$\bar{\alpha }j2015$	0.64	3.12	1.93	2.90	0.76	5.23

AG: Agroforestry; SS: Shrub Savanna; SW: Savanna Woodland; TS: Tree Savanna; OF: Open Forest; CCF: Closed-Canopy Forest; n: number of fragments; Dj: index of dominance;α $\bar{\alpha }$j : mean area

### Drivers of change

**Fig 4:**
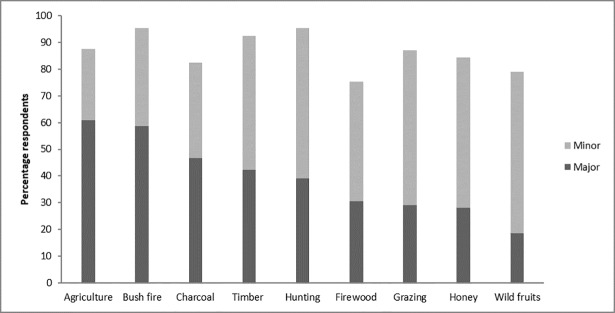
Drivers of change as identified by all respondents.

Participants mentioned that clearing land for agriculture and bush fires were the two main drivers of change in the park (mentioned as important driver by >60% of the participants, Fig 4). Charcoal production and timber extraction were also found to be important drivers, mentioned by >40% of the participants. Interestingly, all drivers considered in this study were reported as ‘a major driver’ by at least some study participants (Fig 4).

**Fig 5:**
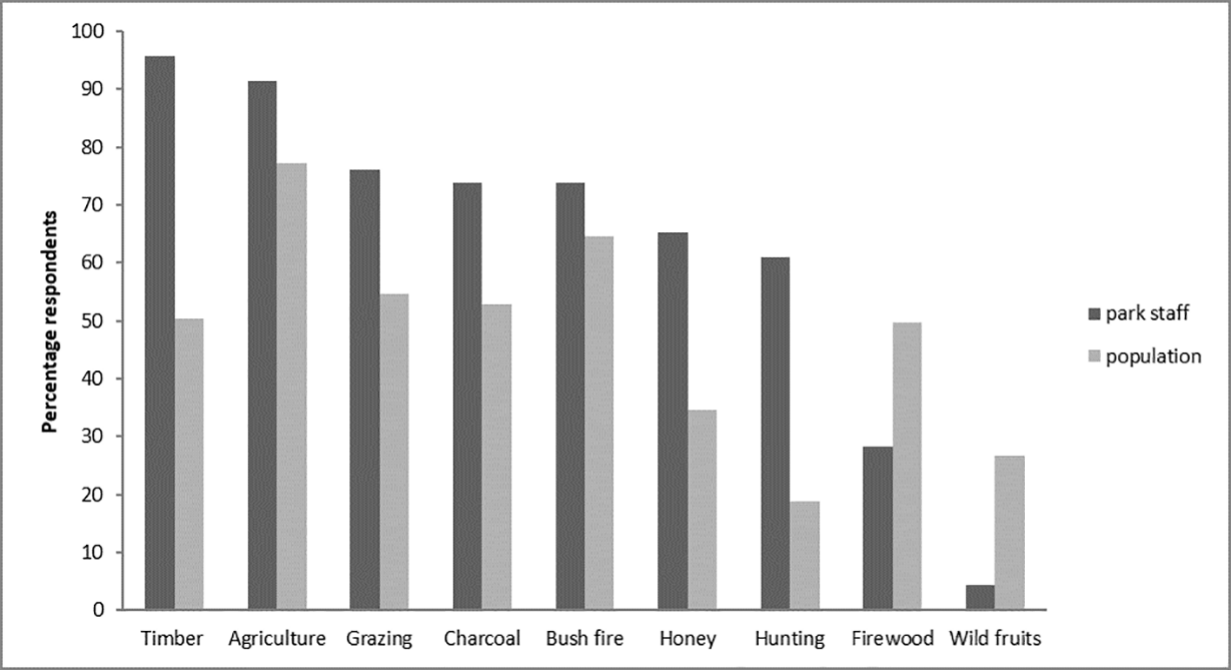
Drivers of change as identified by park staff (managers and rangers) and local populations.

In general, park staff considered most drivers to be more important than local populations, except for firewood and wild fruit harvesting (Fig 5). However, these differences were not significant (p = 0.33). With regard to the different groups within local populations, differences were also observed, with e.g. female farmers considering wild fruits as a more important driver than other groups, or youth considering hunting as an unimportant driver (see S2 Fig in Supplementary information).

Most participants (park staff and populations) linked each driver to a certain vegetation type: e.g. while timber exploitation was a greater issue in closed-canopy forest, clearing land for agriculture was more important in tree-savanna. With regard to the species targeted, for charcoal production these were *Burkea africana*, *Lophira lanceolata* and *Detarium microcarpum* (commonly found in tree savanna and savanna-woodland respectively). The first two species are relatively abundant (see Table S1 in Supplementary information). For firewood, they were *Lophira lanceolata*, *Pterocarpus erinaceus*, *Terminalia laxiflora* and *Crossopteryx febrifuga*, found in different vegetation types. For wild fruits, the preferred species were *Garcinia* spp., *Pentadesma butyracea* and *Detarium senegalense*, found in closed-canopy forest.

For timber, the preferred species was *Pterocarpus erinaceus*, found in closed-canopy forest and savanna-woodland. Park rangers mentioned that the exploitation of this species has considerably increased since 2007, when its value in the international market also increased (see[51]). Two other species targeted for timber were *Milicia excelsa* and *Antiaris toxicaria var*. *africana*, also found in closed-canopy forest only. Unpublished data from the park managers indicates that 4,725 m^3^ of mostly *Pterocarpus erinaceus* was exploited between 2012 and 2015 in this park.

### Comparison with other studies in West Africa

A review of other studies available from West Africa indicates that habitat fragmentation and degradation has also been observed in other countries, both within and outside protected areas (Table 3). In general, a decrease in closed-canopy forest and savanna-woodland, and an increase in agroforestry were observed. The main drivers of change mentioned in these studies were agriculture, wood harvesting, charcoal production and bush fires, like in our study area.

**Table 3:** A review of other studies available from West Africa, including type of study, changes observed, drivers of change and reference.

**Country**	**Location**	**Type of study**	**Changes observed**	**Drivers of change**	**Reference**
Benin	Forest Reserve of Oueme	Focus-groups/questionnaires	increased forest fragmentation,	wood harvesting, bush fires	Sambiéni et al. [52]
Benin	Forest Reserve of Mt Kouffe	Satellite image analysis	decrease in forest, increase in savanna and agroforestry	agriculture	Toko Mouhamadou et al. [53]
Benin	Djidja area	Satellite image analysis and forest inventory	decrease in closed-canopy forest, increase in savanna-woodland	agriculture, charcoal production	Arouna [54]
Benin	Forest Reserve of Mekrou	Documentary research, diachronic analysis of satellite imagery and socio-economic surveys	decrease in natural habitats, increase in agroforestry	firewood harvesting, charcoal and agriculture	Bouko et al. [55]
Burkina Faso	Forest Reserve of Tiogo	Satellite image analysis	decrease in savanna-woodland, increase in tree-savanna and shrub savanna	wood harvesting, agriculture	Tankoano et al.[56]
Burkina Faso	Forest Reserve of Bontioli	Satellite image analysis and questionnaires	decrease in gallery-forest and savanna	firewood harvesting, charcoal and agriculture	Dimobé et al. [57]
Guinea	Mt Nimba forest-savanna mosaic	Ecological study	forest degradation	agriculture and bush fires	Camara et al. [58]
Ivory Coast	Tanda Department, forest-savanna mosaic	Modelling landscape dynamics	decrease in forest, increase in savanna	agriculture and bush fires	Barima et al.[59]
Ivory Coast	Forest Reserve of Mt Korhogo	Satellite image analysis	increase in closed-canopy forest *	increased forest management	Koné et al. [60]
Ivory Coast	Marahoue National Park	Satellite image analysis and questionnaires	decrease in closed-canopy forest	agriculture	N’Da et al. [61]
**Country**	**Location**	**Type of study**	**Changes oberved**	**Drivers of change**	**Reference**
Mali	Wildlife Reserve of Fina	Satellite image analysis	decrease in savanna-woodland, increase in agroforestry	wood harvesting, charcoal production	Diallo et al. [62]
Niger	W National Park	Satellite image analysis	decrease in closed-canopy forest, increase in open forest	bush fires	Inoussa et al. [63]
Togo	Wildlife Reserve of Abdoulaye	Satellite image analysis and forest inventory	decrease in closed-canopy forest and savanna-woodland, increase in agroforestry	wood harvesting, agriculture	Djiwa [64]
Togo	Oti-Kéran and Oti-Mandouri Reserves	Literature review and questionnaires	decrease in natural habitats, increase in agroforestry	agriculture, grazing for livestock	Guelly and Segniagbeto [65]
Togo	Togodo National Park	Satellite image analysis	decrease in closed-canopy forest and savanna-woodland, increase in agroforestry	agriculture, charcoal production, canoe production	Kemavo [66]
Togo	Mono River Basin	Satellite image analysis	increase in farmlands and savanna, decrease in woodlands	Non-available	Diwediga et al. [31]

## Discussion

### Observed changes in vegetation types and drivers

In the past decades, great changes have been observed in the different vegetation types of Fazao-Malfakassa National Park. Overall, there has been a decrease in closed-canopy forest and tree-savanna, an increase in agroforestry, savanna-woodland and shrub savanna, and little change in open forest. Moreover, all vegetation types have become more fragmented. The drivers of change differed between vegetation types.

Closed-canopy forest decreased in area and became increasingly fragmented over time, particularly from 2001 to 2015. This was related to timber exploitation in this vegetation type. Three timber species (*Pterocarpus erinaceus*, *Milicia excelsa* and *Antiaris toxicaria*) are of high commercial value and are logged by local communities. Once these canopy trees are removed, it is likely that the structure of the forest changes: there is more light availability, more grass in the understory, greater biomass for burning and therefore, more fires, further changing the structure and composition of this forest type. Barima et *al*., [59] reported this process in the forest-savanna zone of Ivory Coast. Cochrane et *al*., [67], studying interactions between habitat fragmentation in evergreen tropical forests in Brazil, also reported how increased timber exploitation increased forest vulnerability to fire. Apart from opening the canopy, selective timber exploitation also leaves considerable deadwood biomass (branches, leaves, etc) which are prone to burning [68]. A decrease in closed-canopy forest has also been observed in Abdoulaye Fauna Reserve in Togo [64] and in several other countries in the region, often linked with wood harvesting and clearing land for agriculture (see Table 3). The slight increase in closed-canopy forest between 1987–2001 should be further explained. Before 2001 (especially before 1991), park management was highly repressive, in a military manner, which limited illegal activities such as bush fires set up by hunters (which became more common afterwards).

Tree-savanna also decreased in area and became increasingly fragmented over time. In this case, however, clearing land for agriculture was the main driver of change. Tree-savanna, often dominated by *Terminalia macroptera* and *T*. *laxiflora*, are generally located in flat areas, which are not well-drained [18]. They support an important biomass of grass, making them a preferred feeding site for buffaloes and hartebeest [69], and therefore, carnivores. They are also located at the preferred sites for agriculture. In Tiogo in Burkina Faso, where famers also practice slash-and-burn agriculture, clearing land for agriculture is also the main driver of change in the tree-savanna [56].

The area of agroforestry significantly increased over time. For the first period (1987–2001) this was likely to be related to the socio-political problems of Togo in the early 1990s, when landless people encroached several protected areas [16]. The increase in agroforestry was greater in the eastern part of the park, which is more accessible. A similar situation has been reported from Ivory Coast: following political instability, populations encroached Marahoué National Park [70]. In our study area, the increase in agroforestry was less severe for the period 2001–2015, which might be related to greater elephant presence in the eastern part of the park. Preliminary observations suggest that increased elephant presence in the area is related to more elephants from the nearby Kyabobo National Park (Ghana) staying longer periods of time in our study area, due to decreased hunting pressure in Ghana (unpublished data). Elephants destroy crops such as yams, which discourage farmers from farming inside the park.

Savanna-woodland also increased in area over time. In this case, this might be related to degraded closed-canopy and open forest becoming this habitat type, as in FMNP this vegetation type is located between forests and other savannas. An increase over time in savanna-woodland was also observed in central Benin [54]. Notably, this was not the case in in Tiogo Forest Reserve in Burkina Faso and in Fina Wildlife Reserve in Mali [56,62], where a decrease in savanna-woodland was observed. In drier locations of the forest-savanna mosaic, where there is little closed-canopy forest, savanna-woodland seems to be the habitat type targeted for wood harvesting, agriculture and bush fires.

Shrub savanna also increased in area over time. Interestingly, this might be related to another factor: decreased soil quality in rocky hills related to climatic changes. In the rocky areas of the forest-savanna mosaic, where soils are shallow and infertile, shrub savannas tend to be the dominant vegetation type [71,72]. The shallower and more infertile the soil, the greater the number of shrubs (e.g. in Mt Nimba in Guinea [72]). Because of prolonged droughts, and heavy rains afterwards, soil quality might have decreased in certain parts, which seems to have promoted more shrub growth. Climatic changes have already been observed in Togo, with an increase in mean annual temperature between 0.7 and 1.2°C (compared with the period 1961–1985) and a decrease in annual rainfall between 3 and 81 mm [73]. However, more research is needed to confirm if this increase in shrub savanna is related to climatic and soil changes.

Open forest did not change in area over time, although they became more fragmented. This is different from observations in Ivory Coast, where open forest significantly decreased between 1986 and 2000 [74]. In W National Park in Niger, they increased over time [63]. In our study area, open forest is less affected by human activities than other vegetation types, as (i) the dominant tree species in this forest type (*Isoberlinia* spp.) are not sufficiently harvested for timber [26]; and (ii) grass is not abundant in the understory, which makes this vegetation type less prone to bush fires (pers. Obs.). It has been suggested that under little human disturbance, open forest tend to reach a stable state in the forest-savanna mosaic [75], which might be happening in our study area.

### Consequences of the observed changes

The observed changes in vegetation types in FMNP are likely to have negative effects on biodiversity, humans and carbon stocks. With regard to biodiversity, a decrease in tree-savanna is detrimental for wildlife, as this habitat type is preferred by large ungulates [69]. A decrease in closed-canopy forest is also detrimental for wildlife, in particular for forest elephants, primates and forest birds. It has been shown that increased fragmentation of closed-canopy forest has a negative impact on primates (e.g. in Brazil, as smaller forest patches tend to be inhabited by smaller animal populations, which are at higher risk of extinction [76]). Increased forest fragmentation also makes species more susceptible to climatic changes. Carr et al. [11] highlighted that large forest birds such as *Accipiter erythropus* and *Tauraco persa*, found in FMNP, are particularly vulnerable to climate change. Apart from wildlife, increased forest fragmentation could also negatively affect several shade-tolerant tree species, which might face difficulties to regenerate in environments with more light. One example could be *Antiaris toxicaria* var. *africana* which cannot regenerate in canopy gaps [77].

With regard to humans, a decrease in closed-canopy forest means reduced availability of the ecosystem services they provide. For instance, *Berlinia grandiflora*, a tree only found in riverine closed-canopy forest, is a preferred habitat for bees, and therefore helps provide honey. *Pentadesma butyracea* and *Detarium senegalense*, trees which provide fruits highly appreciated by local populations (which can be traded and are a source of income), are also only found in closed-canopy forest. Apart from that, a decrease in tree-savanna can also be detrimental for humans, as this vegetation type is used for livestock grazing during extreme drought events by the semi-nomadic Peuhl or Fulani pastoralists (pers. Obs.).

With regard to carbon storage, closed-canopy forest tends to have higher carbon stocks than other vegetation types [78]. Tree-savanna also stores important quantities of carbon, particularly in the soil [79]. If the areas of these two habitats, which are probably the ones with higher carbon storage, are decreasing, the national park is also loosing carbon (further research on carbon storage is being undertaken by the authors).

### Improved management interventions

Our results show how considerable vegetation changes have taken place in FMNP. Illegal activities continue, and if we are to conserve the existing mosaic of habitats, which is of high importance to large mammals [80], and humans, more should be done towards it. First, there should be greater law reinforcement so that illegal activities such as charcoal production and timber extraction are minimised. Given the limited number of park rangers, one option could be to involve local communities, as it has been successfully done in Pendjari National Park in Benin [81]. These authors reported a significant decrease in illegal activities since local communities were involved in reporting them.

Local communities could also be more involved in fire and grazing management, as it has been done in W National Park, a transboundary park in Niger, Benin and Burkina Faso [82]. The reduction of illegal bush fires set up by hunters is key if closed-canopy forest fragments are to survive. Studies from W National Park and Marahoue National Park in Ivory Coast show how increased fire control, increased law reinforcement and forest restoration (in degraded areas using e.g. cashew trees) has helped increase forest cover [61,63]. As highlighted by PACO/IUCN [83], greater local governance and locals’ involvement in protected area management can help reverse the observed deforestation and degradation trends in the savanna-forest mosaics of West Africa.

Another management strategy we suggest is the classification of Fazao-Malfakassa National Park into three zones [84]: core, transition and peripheral, with different human activities allowed in each zone. The latter zone would include the agroforestry systems already inhabited by humans, in which agricultural productivity could be increased. This classification would not conflict with the requirements of the UNESCO World Heritage site program [85]. Our suggestion is different from the idea of reducing the area of the park by excluding agricultural land. We suggest that by including agricultural land in the park, the State could have greater control of what is happening to it, and it would also help control human-wildlife conflict (e.g. elephants raiding crops). In Bia Biosphere Reserve in Ghana park zoning was put in place in 2007, with some activities allowed in the peripheral zone (such as mushroom cultivation and livestock grazing)[86]. Thanks to this zoning, between 2007–2009 illegal hunting was significantly reduced and elephant populations in the core zone increased from 45% to 78% [86].

## Conclusions

In the past decades, FMNP has lost 40% of closed-canopy forest and 20% of tree-savanna habitats, and those remaining have become severely fragmented, like all other habitat types in this park. These changes were driven by different factors depending upon habitat type and time period studied. Quantifying and understanding vegetation changes and the drivers of change is crucial if we are to establish improved *informed* management interventions. The approach presented here to assess vegetation changes and drivers of change is easily replicable, and could be used to help inform management decisions elsewhere in the region. We call for more research on land use change in and outside protected areas in the West African forest-savanna mosaic, if we are to meet the challenges of increasing pressures on these important ecosystems, for both biodiversity and humans.

## Supporting information

S1 Fig Location of all the studies we reviewed. (TIF)

S2 Fig Drivers of change as identified by the different groups of respondents. M refers to male and F to female farmers.
(TIF)

S1 Table Main characteristics of the different vegetation types studied, including dominant species, canopy height and percentage of canopy cover (All photos were taken by Atsri K. Honam). (DOCX)

S2 Table Classification accuracy for Landsat 5 TM (January 1987). (DOCX)

S3 Table Classification accuracy for Landsat 7 ETM (December 2001). (DOCX)

S4 Table Classification accuracy for Sentinel-2 (December 2015). (DOCX)

S5 Table Scientific, common and local name of all the species (plants and animals) used in main text. Local names are in Tem, Bassar and Kabyè languages.
(DOCX)

S6 Table Data. (XLSX)
