# Supplementary figures and images for: Changes in the West African forest-savanna mosaic, insights from central Togo
Source: PLoS One. 2018 Oct 5;13(10):e0203999. doi: 10.1371/journal.pone.0203999 (PMC6173393; doi:10.1371/journal.pone.0203999)

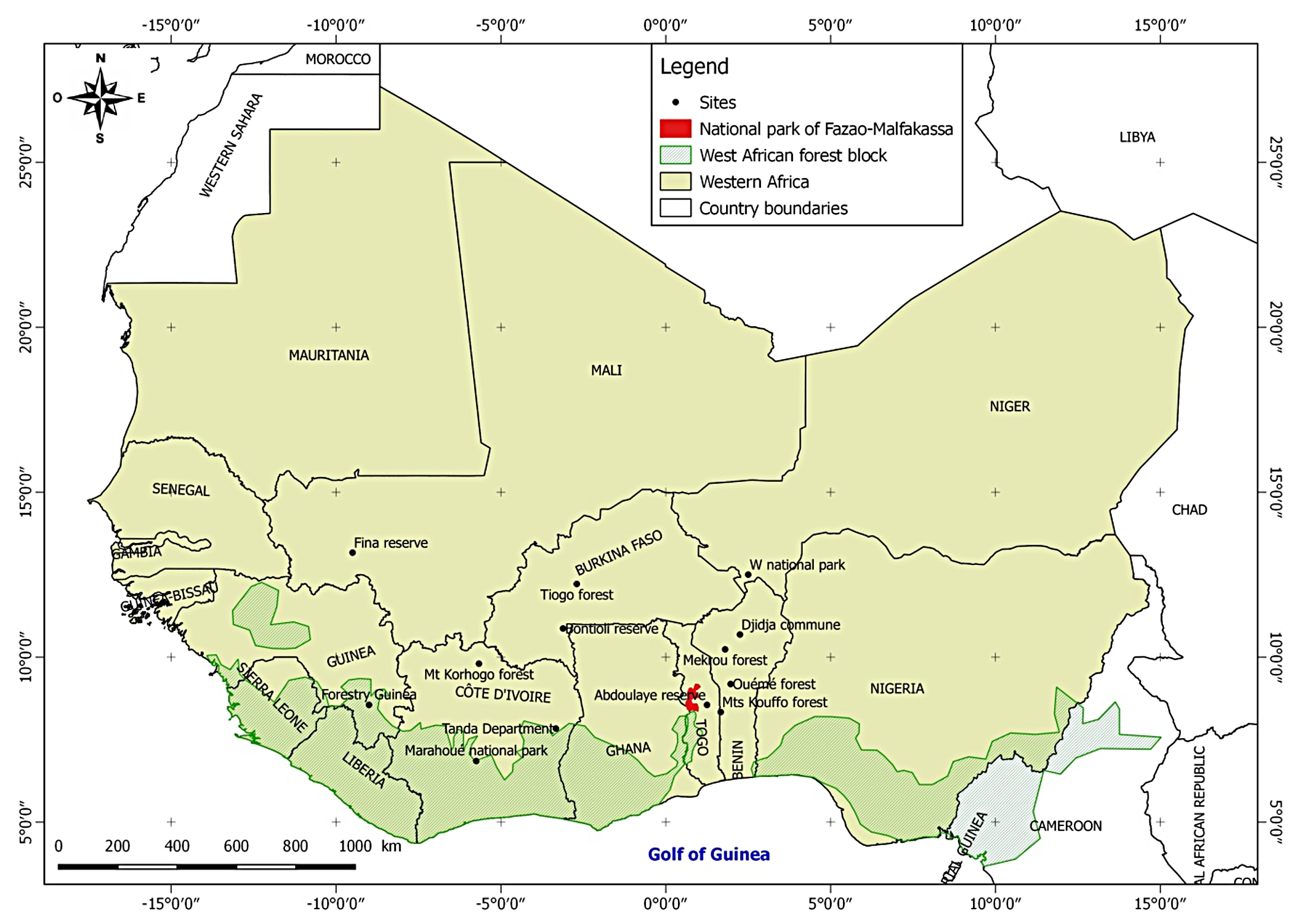

Supplement: S1 Fig — (TIF) [file pone.0203999.s001.tif]

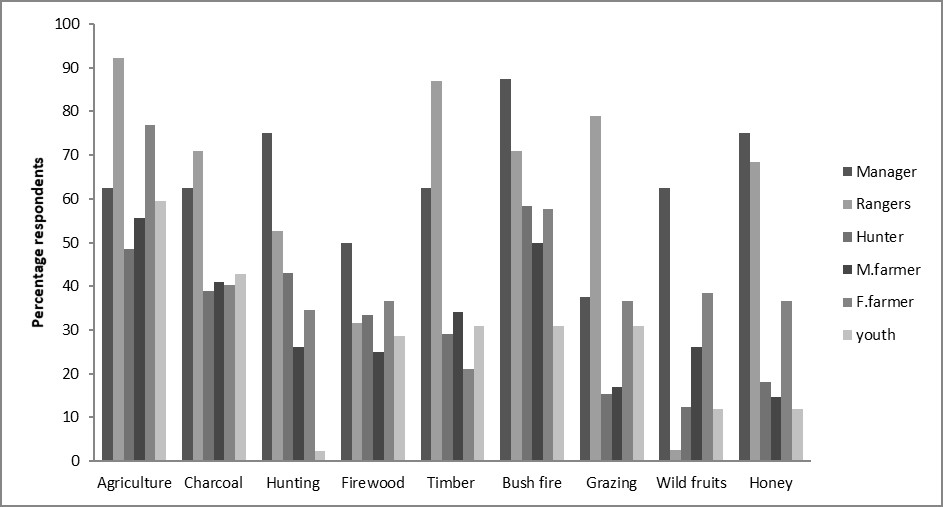

Supplement: S2 Fig — M refers to male and F to female farmers. (TIF) [file pone.0203999.s002.tif]
