# Supplementary material for: Changes in the West African forest-savanna mosaic, insights from central Togo
Source: PLoS One. 2018 Oct 5;13(10):e0203999. doi: 10.1371/journal.pone.0203999 (PMC6173393; doi:10.1371/journal.pone.0203999)
Supplement: S1 Table — (DOCX) [file pone.0203999.s003.docx]

**S1Table.** Main characteristics of the different vegetation types studied, including dominant species, canopy height and percentage of canopy cover (All photos were taken by Atsri K. Honam)

| Land cover | Dominant tree species | Height (m) | Percentage of canopy cover | Photo |
| --- | --- | --- | --- | --- |
| Shrub savanna | *Parinari curatellifolia*, *Crossopteryx febrifuga,* and *Burkea africana* | 2 - 6 | 10-20 % | 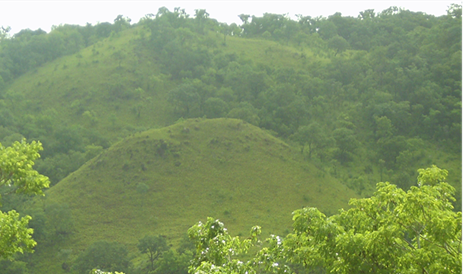 |
| Tree savanna | *Vitellaria paradoxa*, *Terminalia laxiflora, Pteleopsis suberosa*, *Parinari curatellifolia* and *Terminalia macroptera* | 2 - 7 | 20-50 % | 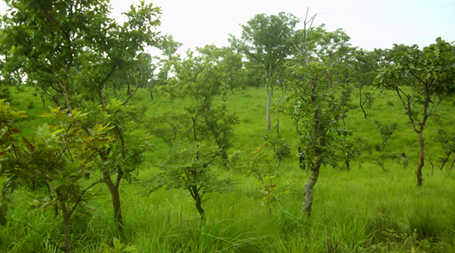 |
| Savanna woodland | *Pseudocedrela kotschyi*, *Lophira lanceolata*, *Terminalia laxiflora*  and *Crossopteryx febrífuga* | 4 - 13 | 40-60 % | 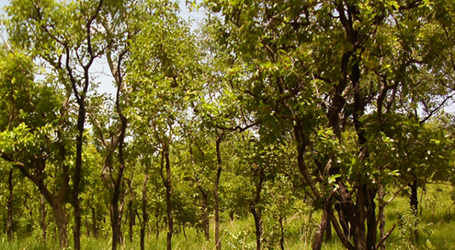 |
| Agroforestry | *Daniellia oliveri, Vitellaria paradoxa*, *Terminalia glaucescens* and *Parkia biglobossa* | 6 - 12 | 20-45 % | 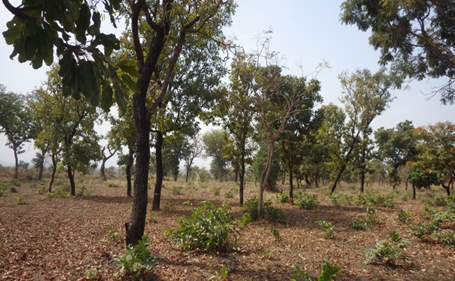 |
| Open forest | *Isoberlinia doka*,  *Isoberlinia tomentosa,* *Monotes kestingii* and *Uapaca togoensis* | 6 - 16 | 50-70 % | 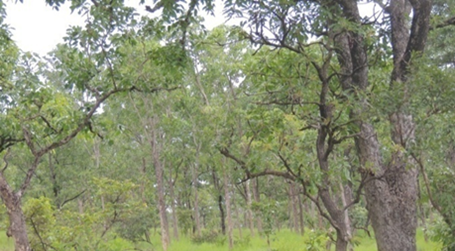 |
| Closed-canopy forest | *Cola gigantea*, *Parinari glabra, Aubrevillea kerstingii* and *Eriocaulon kestingii* | 8 - 20 | 60-90 % | 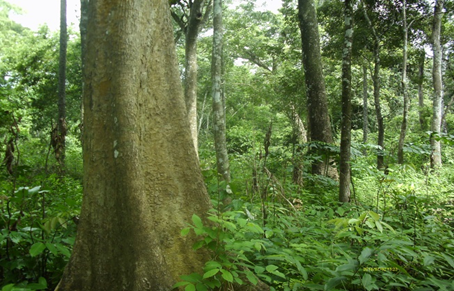 |
