# Supplementary material for: Changes in the West African forest-savanna mosaic, insights from central Togo
Source: PLoS One. 2018 Oct 5;13(10):e0203999. doi: 10.1371/journal.pone.0203999 (PMC6173393; doi:10.1371/journal.pone.0203999)
Supplement: S2 Table — (DOCX) [file pone.0203999.s004.docx]

**S2 Table.** Classification accuracy for Landsat 5 TM (January 1987)

| **Classified** | **Reference** | | | | | |
| --- | --- | --- | --- | --- | --- | --- |
|  | **Closed-canopy forest** | **Open forest** | **Tree savanna** | **Savanna-woodland** | **Shrub savanna** | **Agroforestry** |
| **Closed-canopy forests** | **85.29** | 0 | 0 | 0 | 0 | 0 |
| **Open forest** | 0 | **91.95** | 0.9 | 0 | 1.24 | 0 |
| **Tree savanna** | 14.58 | 6.9 | **97.95** | 4.29 | 0 | 0 |
| **Savanna-woodland** | 0 | 1.15 | 1.15 | **95.71** | 0 | 0 |
| **Shrub savanna** | 0.13 | 0 | 0 | 0 | **89.75** | 2.94 |
| **Agroforestry** | 0 | 0 | 0 | 0 | 9.01 | **97.06** |
| **Total** | 100 | 100 | 100 | 100 | 100 | 100 |
| **Overall accuracy** | 0.98 |  |  |  |  |  |
| **Kappa coefficient** | 0.98 |  |  |  |  |  |
