# Supplementary material for: Changes in the West African forest-savanna mosaic, insights from central Togo
Source: PLoS One. 2018 Oct 5;13(10):e0203999. doi: 10.1371/journal.pone.0203999 (PMC6173393; doi:10.1371/journal.pone.0203999)
Supplement: S3 Table — (DOCX) [file pone.0203999.s005.docx]

**S3 Table.** Classification accuracy for Landsat 7 ETM (December 2001)

| **Classified** | **Reference** | | | | | |
| --- | --- | --- | --- | --- | --- | --- |
|  | **Closed-canopy forest** | **Open forest** | **Tree savanna** | **Savanna-woodland** | **Shrub savanna** | **Agroforestry** |
| **Closed-canopy forest** | **83.13** | 3 | 0 | 0 | 0 | 0 |
| **Open forest** | 0 | **87** | 4.9 | 5 | 1.24 | 2 |
| **Tree savanna** | 7.58 | 3.9 | **85.15** | 4.29 | 0 | 0 |
| **Savanna-woodland** | 7 | 3.15 | 5.95 | **85.71** | 0 | 0 |
| **Shrub savanna** | 2.29 | 2.95 | 4 | 5 | **87.75** | 7.94 |
| **Agroforestry** | 0 | 0 | 0 | 0 | 11.01 | **90.06** |
| **Total** | 100 | 100 | 100 | 100 | 100 | 100 |
| **Overall accuracy** | 0.82 |  |  |  |  |  |
| **Kappa coefficient** | 0.85 |  |  |  |  |  |
