# Supplementary material for: Changes in the West African forest-savanna mosaic, insights from central Togo
Source: PLoS One. 2018 Oct 5;13(10):e0203999. doi: 10.1371/journal.pone.0203999 (PMC6173393; doi:10.1371/journal.pone.0203999)
Supplement: S4 Table — (DOCX) [file pone.0203999.s006.docx]

**S4 Table.** Classification accuracy for Sentinel-2 (December 2015)

| **Classified** | **Reference** | | | | | |
| --- | --- | --- | --- | --- | --- | --- |
|  | **Closed-canopy forest** | **Open forest** | **Tree savanna** | **Savanna-woodland** | **Shrub savanna** | **Agroforestry** |
| **Closed-canopy forest** | **91.56** | 3.89 | 0 | 11.42 | 1 | 0 |
| **Open forest** | 5.35 | **79.26** | 18.18 | 5.72 | 0 | 0 |
| **Tree savanna** | 2.97 | 4.88 | **81.82** | 8.57 | 0 | 0 |
| **Savanna-woodland** | 0.12 | 6.61 | 0 | **74.29** | 0 | 0 |
| **Shrub savanna** | 0 | 0 | 0 | 0 | **96** | 5.5 |
| **Agroforestry** | 0 | 5.36 | 0 | 0 | 3 | **94.5** |
| **Total** | 100 | 100 | 100 | 100 | 100 | 100 |
| **Overall accuracy** | 0.97 |  |  |  |  |  |
| **Kappa coefficient** | 0.90 |  |  |  |  |  |
