# Supplementary material for: Changes in the West African forest-savanna mosaic, insights from central Togo
Source: PLoS One. 2018 Oct 5;13(10):e0203999. doi: 10.1371/journal.pone.0203999 (PMC6173393; doi:10.1371/journal.pone.0203999)
Supplement: S5 Table — Local names are in Tem, Bassar and Kabyè languages. (DOCX) [file pone.0203999.s007.docx]

**S5 Table.** Scientific, common and local name of all the species (plants and animals) used in text. Local names are in Tem, Bassar and Kabyè language.

| **Scientific name** | **Common name** | **Local name** | **Type of species** |
| --- | --- | --- | --- |
| *Afzelia africana*. Sm. & Pers. | Lucky-bean tree | Welu (Tem)/Dkpande (Bassar) | Plant |
| *Antiaris toxicaria* var. *africana* Scott-Elliot ex A.Chev. | Bark Cloth Tree | Doulu (Tem) | Plant |
| *Berlinia grandiflora* (Vahl) Hutch. & Dalziel | Red oak | Boudawo (Tem)/Tabo (Kabyè) | Plant |
| *Burkea africana* Hook. | Wild seringa | Dinaglind (Bassar)/Akagniou (kabyè) | Plant |
| *Cordia platythyrsa* Baker | African *Cordia* |  | Plant |
| *Crossopteryx febrifuga* (Afzel. ex G.Don) Benth. | African bark | Kizame (Tem) | Plant |
| *Detarium microcarpum* Guill. & Perr. | Sweet detar | Dinakpoukr (Bassar) | Plant |
| *Detarium senegalense* J.F.Gmel. | Tallow Tree | Agbankabia (Tem) | Plant |
| *Khaya grandifoliola* C.DC. | African mahogany | Formou (Tem) | Plant |
| *Khaya senegalensis* (Desv.) A.Juss. | African mahogany | Formou (Tem) | Plant |
| *Lophira lanceolata* Tiegh. ex Keay | Dwarf Red Ironwood/false shea | Tchignili (Tem) | Plant |
| *Milicia excelsa* (Welw.) C.C.Berg | Iroko | Doulu (Tem) | Plant |
| *Pararistolochia goldieana* (Hook.f.) Hutch. & Dalziel | kotoku saabore |  | Plant |
| Parkia biglobosa (Jacq.) G.Don | African locuste bean tree | Sulu (Tem)/Boudao (Bassar) | Plant |
| *Pentadesma butyracea* Sabine | Butter Tree | Agbététewou (Tem) | Plant |
| *Pouteria alnifolia* (Baker) Roberty |  |  | Plant |
| *Pterocarpus erinaceus* Poir. | African Barwood/African teak | Temou (Tem) | Plant |
| *Terminalia laxiflora* Engl. |  | Sowu (Kabyè) | Plant |
| *Terminalia macroptera* Guill. & Perr. |  | Sowu (Kabyè) | Plant |
| *Vitellaria paradoxa* C.F.Gaert | Shea | Somu (Tem) | Plant |
| *Accipiter erythropus* (Hartlaub, 1855) | Red-thighed sparrowhawk | Ilim (Tem)/Maquiè (Kabyè) | Animal |
| *Alcelaphus buselaphus* (Pallas, 1766) | Hartebeest | Sougbamo (Tem) | Animal |
| *Colobus vellerosus* (Geoffroy, 1834) | Black-and-white *colobus* | Ninva (Tem) | Animal |
| *Fraseria cinerascens* ( Hartlaub, 1857) | White browed Forest Flycatcher | Cinka (Tem)/soumo (Kabyè) | Animal |
| *Galago senegalensis* (Geoffroy Saint-Hilaire, 1796) | Senegal *Galago* | Ninva (Tem) | Animal |
| *Loxodonta africana* (Blumenbach, 1797) | Savanna elephant | Tou (Tem) | Animal |
| *Loxodonta cyclotis* (Matschie, 1900) | Forest elephant | Tou (Tem) | Animal |
| *Panthera leo* (Linnaeus, 1758) | Lion | Gouni (Tem)/Toyou (Kabyè) | Animal |
| *Perodicticus potto* (Müller, 1766) | Potto | Ninva (Tem) | Animal |
| *Syncerus caffer* (Sparrman, 1779) | African buffalo | Fonowou (Tem)/tènowou (Kabyè) | Animal |
| *Tauraco persa* (Linnaeus, 1758) | Guinea turaco | Douvoré (Tem)/ohoyé (Kabyè) | Animal |
